# Intelligence

**Published:** 1832-04

**Authors:** 


### INTELLIGENCE.

### CHOLERA IN LONDON.

Since the publication of our last number, we have taken every opportunity of obtaining information relating to the existence of Cholera in the metropolis: we have seen many of the cases in the Borough, St. Giles's, and St. Marylebone, and, as far as we are capable of judging, the disease now existing in the metropolis is identically the same as that which has been described as the Indian cholera. There are still, however, some medical practitioners, (most of them, indeed, of little experience and little authority,) who assert that the disease now prevailing in London is not new, and that it is not cholera. Where and when they have witnessed a similar disease, they do not condescend to tell us; and what it is, if it be not cholera, they do not venture to declare. Amongst other "facts" that have been stated by the "no-cholera" party, it has been asserted that the Bills of Mortality for London prove that the number of deaths has not been increased by the disease: but this assertion is in direct opposition to the fact; for the Bills of Mortality shew that, where the cholera has existed, the mortality has been greatly increased,* and where it has not existed, the average number of

* At a recent meeting of the Common Council of the city, it was stated by a member that the number of deaths was now 451 a week in the cholera districts, while the former average number of deaths was only 250.

deaths is about the same as it has usually been at this period of the year. We do not mean to accuse those who are opposed to us of dishonesty, or of being actuated by any interested motives: they have arrived at their erroneous conclusions by consulting the Bills of Mortality for the first week or two only after the disease first appeared in London, when its destructive career was just commencing.

We give the following extracts from the fifth number of the Cholera Gazette:

"*Cases communicated by* Mr. ADOLPHUS BARNETT, *House Surgeon to the Cholera Hospital, Limehouse.*

"CASE I. Abraham Pier, aged thirty years, residing at 12, West row, St. Ann's place, Tile yard, was attacked on Tuesday, 6th March, between eleven and twelve P.M., with a feeling of oppression at the chest, and unpleasant feeling in his head; felt very ill through the night; and at five A.M. had severe purging and vomiting.

"Half-past seven A.M. Feels very weak; vomiting and purging still continue; severe cramps in the thighs, legs, and hands; skin of fingers corrugated; countenance sallow; skin moist; pulse quick and weak.

"Ten A.M. Brought to the hospital: hot air applied.

"Eleven A.M. Extremities cold; tongue furred and moist; pulse 128, and very weak; heat of tongue and hands 75° Fahrenheit. To have opium, half a grain, calomel, five grains, every hour; hot air bath; brandy, half an ounce.

"Twelve M. Complains of the heat. Discontinue hot air bath; brandy, half an ounce.

"One P.M. Eyes very much sunken, with a dark halo around them; extremities cold; tongue moist; profuse perspiration; feet blue; cramps in the legs; skin of hands more corrugated; pulse small and weak. Hot air bath; brandy, one ounce.

"Two P.M. Perspiration diminished. Continue calomel and opium; brandy, half an ounce.

"Three P.M. Feels very weak; face and extremities cold; perspiration very much diminished; pupil contracted; pulse 135: wishes for cold water. Continue calomel and opium; brandy, half an ounce; hot air bath.

"Half-past three P.M. Brandy, one ounce.

"Four P.M. Voice scarcely audible; extremities cold; pulse 130; pupil very much contracted; breath cold; respiration hurried; dejections passing involuntarily. Continue calomel and opium; brandy, half an ounce; hot air bath.

"Five P.M. Extremities and face cold; eyes suffused; breathing more difficult. Four ounces of blood taken; continue brandy, calomel, and opium; hot air bath.

"Feels relieved by the bleeding; pulse 120.

"Half-past five P.M. Unable to speak; pulse scarcely perceptible; extremities cold; pupil very much contracted. Brandy, half an ounce.

"Six P.M. No pulse; pupil much dilated.

"Half-past six P.M. Pupil contracted; respirations five in the minute. Died at seven P.M.

"*Note.* The hot air bath was applied at one P.M., with a view to check the perspiration, and that it had the desired effect will be seen by the subsequent report of two and three P.M.—Attended by T. W. BARNETT, Surgeon, Limehouse.

"CASE II. Julia Carr Mahoney, aged thirty, was attacked on Tuesday, February 28th, with purging and vomiting, which continued until Friday morning, when she had severe cramps in the legs. To take half a grain of calomel, five grains camphor.

"Nine P.M. Removed to the hospital. Hot air bath; continue calomel and opium.

"Eleven P.M. Complains of great debility, and of cramps in the legs; eyes sunken; face cold; tongue appears as if covered with chalk; pulse very small and weak. Mustard poultice to stomach; continue calomel and opium.

"Twelve. Complains very much of poultice; has vomited almost every five minutes since last report; countenance anxious; extremities cool. Continue calomel and opium.

"Half-past eight A.M. Says she feels better; had a stool about four o'clock, like dirty water; vomited at five; extremities warm; pulse ninety-five; feels no pain, but wishes the poultice to be removed; has had cramps three or four times during the night, and wished to be rubbed, which was accordingly done. Continue calomel and opium.

"One P.M. Has been retching very much since last report; complains of heartburn; it is with great difficulty we can get her to answer questions put to her; extremities warm; pulse very weak; tongue furred and moist; pain on pressure at epigastrium; has vomited a small quantity of fluid, like rice water; bladders of warm water to feet and stomach.

"Three P.M. Feels very weak; pulse eighty and stronger; has had a stool of a dark green colour. Continue calomel and opium.

"Ten P.M. Feels much the same as at last report; desires to have her drink given to her with a spoon, she is so very weak; pulse 104; tongue not so furred. Continue calomel and opium.

"4th, two P.M. Feels much the same; tongue furred and moist; pulse ninety-four, and soft; has taken a dose of castor oil, since which she has vomited; she retained on her stomach whatever she took, viz. beeftea, gruel, &c. To take sulphate of quinine, ten grains; aromatic confection, two drachms; pimento water, eight drachms; two large spoonsful every second hour.

"5th, eleven A.M. Feels very weak; bowels have been acted on five or six times since last report, the last stool being bilious; vomiting still continues, but not so frequent. Continue mixture.

"6th, ten A.M. Says she feels no pain; asks continually to be raised up in bed; extremities cold; livid appearance of skin; breathing very difficult; no pulse at the wrist; conjunctiva injected; wishes the bedclothes to be removed. Mustard poultices to stomach, back, calves of legs, and feet.

"Four P.M. Unable to speak, but appears perfectly sensible; pulse scarcely to be felt in axilla; extremities cold; skin shrivelled. Died at five P.M.

"Attended by Mr. CUMMINS, Surgeon, Limehouse."

"*Report on the Cases in the 'Taylor' Family, Bull's Head court, Kent street.*

"*Albany Road; March* 5, 1832.

"Sir: It is with feelings of no ordinary pain that I have to lay before you the annexed details of a series of cases which have occurred in this district, and which are remarkable, as well for the desolation occasioned in one family, as for the rapidity, unexampled in this kingdom, with which the fatal event took place in one of the cases. The collateral circumstances, it will also be seen, are of some interest:

"CASE I. William Taylor, aged fifty, residing in Bull's Head court, Kent street, was seized with the usual symptoms of malignant cholera, on the 29th of February, at two A.M. The disease ran its usual course, and he died at nine A.M., the next day.

"CASE II. Mary Taylor, aged thirteen, the daughter of No. 1, residing in the same house, was seized at noon, on the day of her father's death, 1st March, and died the next day, after an illness of twenty-four hours.

"CASE III. Mrs. Taylor, aged about thirty-five, remained in perfect health during the illness of her husband and daughter, both of whom she attended. At three P.M., on Saturday, 3d of March, she was accidentally seen by Mr. Boddy, the parish surgeon, and found to be in good apparent health, and 'looking very well.' A few minutes before five P.M., an inmate of the same house, while coming up stairs, heard a heavy noise in Mrs. Taylor's apartment, and on entering found that the poor creature had been seized with the disease, while standing by the fire place, and had actually been struck down so unexpectedly, that she fell into the fire. Purging and vomiting of the characteristic cholera fluid supervened in a few minutes, and with extraordinary violence; she became perfectly cold and pulseless, but was comparatively unaffected by cramp. At half-past nine P.M., she was placed in a reclining chair, and conveyed with every care to the Cholera Hospital, in York street, Newington. The bearers arrived at a few minutes to ten; she was conveyed to bed, but died in a quarter of an hour, having been but five hours and a quarter ill.

"CASE IV. Thomas Taylor, aged three, son of the deceased Mrs. Taylor, lay in good health by his mother's side, until she was removed to the hospital. This morning, March 4, he was attacked in the usual manner, and was in a desperate state when I visited him at the hospital, at two P.M.*

"When I state that, in the same house wherein the preceding cases have occurred, there were at the time of the first seizure, and are up to this moment, residing ten other individuals unconnected with the Taylors, and all of whom are as yet free from the disease; when I add, that the deceased persons occupied the second floor, while the ground floor was tenanted by individuals yet unaffected; and lastly, when I point to the sequence of periods in the occurrence of the cases, I need not labour much to shew how utterly inconsistent is such a train of events and circumstances with the laws and operations of all diseases of aerial or terrestrial origin.

"Regretting much the occasion which has called for the present communication, I am, Sir, &c. W. B. O'SHAUGHNESSY, M.D."

"W. MACLEAN, Esq."

The report from the Central Board of Health to the 29th of March shews the number of cases in the metropolis since the commencement of the disease, to be 1,665; deaths, 881. We do not think it necessary to give the details of the report, as they are contained in every newspaper in the kingdom.

---

CHOLERA. *Address to the Medical Profession, and to the Public in general.*

THE present alarm respecting the disease which is now said to exist in London and its vicinity, under the name of Cholera, is maintained by some to be unfounded or exaggerated; while by others it is asserted that this alarm is neither without cause, nor disproportionate to the danger. Whence such diversity of opinion may have originated, has not been ascertained; nor has it been discovered whether there be any reasonable cause for the prevalence of opinions so opposed.

The unsettled state of the public mind has been productive of consequences the most pernicious: our national relations have been injured, our internal prosperity has been shaken. The medical profession have been aspersed by one party, as encouraging panic without cause; and by another, as concealing from the public eye the real nature and the full extent of the disease.

To clear the character of the medical faculty of these aspersions, by inquiring into the actual causes of this general alarm, and to reconcile these conflicting opinions, a Medical Association has been formed, to investigate the nature, modes of propagation, extent, and treatment, of that disease, and lay candidly before the public the results of its inquiries. As this Association belongs to no exclusive party, it advocates no exclusive views. The practitioners, of whom it is composed, espouse no theory; they support no faction; their bond of union is the public good; their common object is the cause of truth; their interests are promoted neither by encouraging excitement nor by resisting just alarm. If the disease known under the name of cholera exists in London, their object is to ascertain its extent, to elucidate its causes, to explain its management, and to restore the tranquillity of the public by an honest statement of the truth.

* "He died of the secondary affection on the following Thursday."

An Association formed with motives so pure and an object so important, can have no enemies but those of science and of mankind: they, therefore, look to the public for countenance, and to the profession for assistance. They more especially solicit the co-operation of the medical officers of parishes and guardians of the poor. As they intend no opposition, they anticipate no resistance. In the truest spirit of philanthropy they have been organised, and with the most rigid respect to truth and candour will all their labours be conducted. They feel that the principles which they follow can lead them only to truth, and they are persuaded that the plan upon which these principles shall proceed can scarcely fail to ensure ultimate success.

The proceedings of the Association shall at all times be open to inspection; the register for the admission of members is now prepared for signatures; and while they express the most friendly feelings towards such as may decline to join them, the Association earnestly solicit, and confidently hope for the speedy co-operation of all who are zealously attached to the interests of medical science, and wish sincerely to promote the welfare of the human species.

L. STEWART, M.D., Chairman.

---

CHOLERA. ORDER ISSUED BY THE PRIVY COUNCIL.

After continuing the Boards of Health at present constituted, it is added, "And it is further ordered, that every practitioner of medicine, within every city, town, or district, in which every such Board of Health is or shall be constituted, by order of the Lords and others of his Majesty's Privy Council, shall, and he is hereby required and commanded to make to such Board a daily report under his hand, containing a numerical report of all cases, deaths, and recoveries of every person attended by such practitioner, who may be affected with the said disease, or *with any other disease anywise resembling the same.* And all medical practitioners who shall neglect or omit to make any such return at the time, or in the manner or form required by the Board of Health of the city, town, or district, in which they reside, or in which the patient they attend resides, or who shall in such return wilfully make any false statement, are hereby warned and admonished that the penalties and punishments consequent upon any such disobedience to this order, and to the provisions of the before-mentioned Act of Parliament, will forthwith be enforced against him."

---

### MONTHLY LIST OF MEDICAL BOOKS.

### METEOROLOGICAL JOURNAL,

By Messrs. HARRIS and Co. Mathematical Instrument Makers, 50, High Holborn.

| | Moon. | Rain gauge. | Thermometer. | | | Barometer. | | De Luc's Hygrometer. | | Winds. | | Atmospheric Variation. | | |
|---|---|---|---|---|---|---|---|---|---|---|---|---|---|---|
| Feb. | | | 9a.m. | max. | min. | 9a.m. | 10p.m. | 9a.m. | 10p.m. | 9 a.m. | 10p.m. | 9 a.m. | 2 p.m. | 10 p.m. |
| 20 | | | 36 | 40 | 30 | 30.14 | 30.12 | 63 | 65 | SE | ESE | foggy | fine | fine |
| 21 | | | 32 | 38 | 31 | .05 | .06 | 65 | 65 | ESE | ESE | | cloudy | foggy |
| 22 | | | 33 | 43 | 32 | .15 | .19 | 65 | 66 | SE | SSE | fog | foggy | |
| 23 | ☾ | | 32 | 35 | 30 | .16 | .09 | 68 | 69 | SE | SE | | | fog |
| 24 | | | 32 | 34 | 29 | 29.91 | 29.81 | 69 | 71 | SE | SE | | | |
| 25 | | | 33 | 36 | 32 | .90 | 30.00 | 70 | 70 | NE | NE | foggy | | |
| 26 | | | 38 | 41 | 33 | 30.00 | 29.97 | 70 | 70 | E | SE | | fine | fine |
| 27 | | | 36 | 38 | 35 | 29.95 | 30.00 | 70 | 69 | NE | ESE | | cloudy | cloudy |
| 28 | | | 37 | 40 | 35 | 30.00 | .02 | 67 | 67 | SE | NNE | cloudy | | |
| 29 | | | 36 | 39 | 36 | .02 | .04 | 64 | 64 | E | SW | foggy | fine | |
| Mar. 1 | | | 39 | 45 | 40 | .10 | .13 | 62 | 65 | SW | SW | | foggy | sleet |
| 2 | ● | | 43 | 43 | 40 | .14 | .14 | 65 | 64 | SW | SSW | | | cloudy |
| 3 | | | 42 | 45 | 39 | .10 | .01 | 64 | 63 | S | SSW | fine | fine | sleet |
| 4 | | | 44 | 47 | 40 | 29.70 | 29.58 | 65 | 66 | SW | W | cloudy | showery | rain |
| 5 | | .23 | 45 | 47 | 35 | .62 | .85 | 67 | 65 | W | NW | fine | fine | fine |
| 6 | | | 43 | 47 | 35 | .51 | .15 | 64 | 65 | SSW | SW | cloudy | rain | |
| 7 | | .10 | 41 | 44 | 34 | .21 | .19 | 65 | 65 | SW | SW | fine | | cloudy |
| 8 | | | 38 | 43 | 33 | .38 | .60 | 65 | 64 | NNW | WNW | foggy | fine | fine |
| 9 | ☽ | | 40 | 45 | 33 | .89 | 30.08 | 64 | 63 | WNW | ENE | | | |
| 10 | | | 38 | 42 | 35 | 30.20 | .15 | 62 | 62 | ESE | SSE | fog | | |
| 11 | | | 39 | 41 | 34 | .03 | 29.96 | 64 | 65 | SE | SE | foggy | foggy | |
| 12 | | | 38 | 43 | 36 | 29.85 | .81 | 67 | 67 | SE | SE | | fine | |
| 13 | | | 40 | 46 | 40 | .72 | .62 | 67 | 67 | SW | SSW | | | |
| 14 | | | 47 | 49 | 37 | .31 | .16 | 67 | 67 | SSW | S | cloudy | showery | cloudy |
| 15 | | .25 | 42 | 44 | 35 | .26 | .54 | 67 | 66 | NE | SSE | rain | fine | fine |
| 16 | ○ | | 40 | 50 | 43 | .63 | .47 | 65 | 68 | SW | SW | foggy | cloudy | rain |
| 17 | | | 50 | 52 | 38 | .25 | .33 | 69 | 62 | W | WSW | rain | fine | fine |
| 18 | | | 43 | 47 | 40 | .56 | .71 | 62 | 60 | W | W | fine | | cloudy |
| 19 | | .17 | 49 | 50 | 40 | .67 | .56 | 60 | 56 | W | W | | sleet | |

The Rain gauge having been frozen in February, the quantity cannot be given.

